# Cognitive-affective and behavioral pain mechanisms in individuals with chronic low back pain: a network analysis

**DOI:** 10.1097/j.pain.0000000000004020

**Published:** 2026-06-16

**Authors:** Putu Gita Nadinda, Antoinette I.M. van Laarhoven, Lourens J. Waldorp, Johannes W.S. Vlaeyen, Madelon L. Peters, Andrea W.M. Evers

**Affiliations:** a Health, Medical and Neuropsychology Unit, Leiden University, Leiden, the Netherlands; b Experimental Health Psychology, Department of Clinical Psychological Science, Maastricht University, Maastricht, the Netherlands; c Department of Psychological Methods, University of Amsterdam, Amsterdam, the Netherlands; d Research Group Health Psychology, Faculty of Psychology and Educational Sciences, KU Leuven, Leuven, Belgium; e Medical Delta, Leiden University, Technical University Delft, and Erasmus University Rotterdam, Leiden, the Netherlands

**Keywords:** Chronic pain, Networks, Expectancy, Avoidance

## Abstract

Various cognitive-affective and behavioral factors contribute to the maintenance of low back pain, but these factors are often evaluated in isolation. The network approach provides an avenue to evaluate multiple cognitive-affective and behavioral factors at once and across time. This study explored the relationship between pain, expectancies, avoidance, and related cognitive-affective and behavioral factors using network analysis. A total of 30 individuals with chronic low back pain completed ecological momentary assessments regarding their physical symptoms and related cognitive-affective and behavioral factors 5 times per day for a duration of 2 weeks. Temporal and contemporaneous networks were estimated using vector autoregressive models on a group and individual level. Networks show that the relationship among cognitive-affective and behavioral factors reinforces each other within the same time frame as well as across time. The strongest temporal connections were found between pain and attention, while the strongest contemporaneous connections were found between fear and attention at the group level. On an individual level, different variations of network structures can be found, suggesting that a more individualized approach is needed for better treatment outcomes. Further research is warranted to confirm the causal relations between cognitive-affective and behavioral factors in the maintenance of chronic pain. The network approach may be a useful tool to develop more effective individualized treatment to manage chronic pain.

## Abstract 2

Supplemental Digital Content is Available in the Text.

Variations are found in the network structure of cognitive-affective-behavioral factors in individuals with chronic low back pain, indicating the need for a more personalized approach.

## 1. Introduction

Chronic low back pain (CLBP) is a type of chronic primary musculoskeletal pain that persists for at least 3 months and has been associated with a large social and economic burden.^6,12,67^ Chronic low back pain can lead to disability, lower physical functioning, and may affect work productivity (e.g., Ref. 6, 41, 67). In addition, up to 10% of the population have been diagnosed with CLBP with a higher prevalence in individuals with older age.^40^

Of the various biological and psychosocial factors that can influence the perception and maintenance of pain,^20,32^ expectancies and avoidance are believed to be key psychological mechanisms that interact and dynamically change over time.^45^ These 2 mechanisms play a large role in pain perception by maintaining negative beliefs about pain^1,24,45,49,58^ and in the maintenance of both fear of pain and pain catastrophizing, which can often lead to further pain, disability, and movement restriction.^36,62,63^ The dynamic interaction between expectancies and avoidance in pain can also change through the presence of other related cognitive-affective factors. For example, there is evidence that pain avoidance is related to negative mood^18^ and high pain expectancies have been shown to be associated with more bodily attention which have also been shown to affect pain perception.^27^

One way to assess the dynamic interaction between cognitive-affective and somatic symptoms in individuals with CLBP is through a network approach.^64^ Recently, there has been growing interest in using the network approach in the medical and behavioral sciences field.^39,55^ The network approach shifts the perspective from identifying an underlying cause of a condition (e.g., searching for an underlying pathology) to viewing a condition (e.g., chronic pain) as a system of interconnected symptoms and mechanisms.^4,42^ Considering that the direct cause to chronic pain is not always clear and the onset of chronic pain symptoms cannot be easily ascertained, the network approach provides a good avenue to assess the different cognitive-affective and behavioral factors that may be contributing to the maintenance of pain. In fact, this approach has been used to assess the dynamic relationship between psychological factors and somatic symptoms (including pain) using longitudinal data (e.g., Ref. 2, 9, 56), with each focusing on a different aspect of psychopathology (e.g., depression, paranoia) and different target groups (e.g., adolescents with chronic pain, individuals with chronic somatic symptom disorder). Similarly, a growing number studies have investigated this relationship using cross-sectional data in different chronic pain conditions (e.g., Ref. 8, 50, 69). Although there is evidence supporting the theory postulating that the interaction between expectancies, avoidance, and related cognitive-affective and behavioral factors can perpetuate pain, and that pain itself can be a predictor of future pain,^2,47^ to the best of our knowledge, how these factors interact over time within a network in CLBP is not yet known. Therefore, in this study, we aimed to explore the temporal relationship between pain, expectancy, avoidance, and related cognitive-affective and behavioral factors, including fear, attention, and negative affect in individuals with CLBP. Through the network approach, we can assess the dynamic relationship between multiple variables at a group and individual level.^14^

## 2. Methods

This study was an exploratory ecological momentary assessment (EMA) study that was conducted over a duration of 2 weeks. The study was approved by the Leiden University Psychology Research Ethical Committee (2022-12-06-A.W.M. Evers-V1-4376) and has been preregistered through the open science framework (see: https://doi.org/10.17605/OSF.IO/QH79B).

### 2.1. Participants

We aimed to recruit a total of 30 individuals with CLBP. Participants were recruited through general advertisements, social media, word of mouth, and a research registry (Hersenonderzoek.nl). Participants were included in the study if they were 18 to 65 years of age, met the inclusion criteria for primary CLBP based on the ICD-11,^46^ were fluent in Dutch, and had access to the internet and a smartphone device. The exclusion criteria were diagnosed comorbid pain symptoms (e.g., fibromyalgia, arthritis), and use of recreational drugs (e.g., cannabis, XTC) for more than 3 times per month.

### 2.2. Materials and measures

#### 2.2.1. Cross-sectional measures

Participants completed a set of questionnaires assessing demographics data (e.g., age, sex, education level, occupation), duration of CLBP symptoms, pain expectancy over the next 2 weeks, medication use, enrollment in therapy, current pain status (assessed using a 0 [*no pain*] to 10 [*extreme pain*] numerical rating scale), and smoking status.

To measure daily physical activities, participants completed the short form International Physical Activity questionnaire,^3,30^ which is a self-report measure of physical activities that consists of 7 questions measuring the duration of vigorous physical activities (in days per week and minutes per day), moderate physical activities (in days per week and minutes per day), walking (in days per week and minutes per day), and time spent sitting (measured in minutes per day). Time spent doing vigorous physical activities were categorized as high activities, moderate physical activities were categorized as moderate activities, walking was categorized as low activities, and time spent sitting was categorized as sedentary movement.

Furthermore, participants completed the Depression, Anxiety, and Stress Scale (DASS-21^34^), which is a self-report measure of depression, anxiety, and stress that contains a total of 21 questions, and 7 questions for each subscale. Participants were asked to rate how much the statements applied to them over the last week. The total score of the DASS-21 ranges from 0 to 42 for each subscale with higher scores indicating more severity.

In addition, participants completed the Oswestry Disability Index,^19^ which is a self-report measure of pain-related disability consisting of 10 sections. Each section consists of different domains of disability such as pain intensity, personal care, lifting, walking, and standing. Participants were asked to select a statement within each section that best reflected their problem. The total score of the Oswestry Disability Index ranges from 0 to 100, with higher scores indicating more severe disability (e.g., bedbound). These set of questionnaires were asked once at baseline.

#### 2.2.2. Ecological momentary assessment

The ecological momentary assessment (EMA) questions consisted of a total of 14 items relating to symptoms and mechanisms related to CLBP that was measured over the course of 2 weeks (see supplemental digital content, File 1 for a complete list, http://links.lww.com/PAIN/C512). Each item could be answered on a scale from 0 (*none at all*) to 6 (*extremely*). Ten of the items pertained to key factors that have been shown to maintain pain namely: pain intensity, negative expectancy (measured using 2 items; one item to measure stimulus expectancy and the other to measure outcome expectancy^49^), avoidance (measured using 2 items; one relating to avoidance of pain and the other relating to avoidance of back harm), fear (measured over 2 items; one to measure emotional fear of pain [affective component of fear], and the other to measure worry of pain [cognitive component of fear]), attention toward pain, and negative affect (i.e., sadness and stress). One item measured fatigue level—a related somatic symptom that may be present in CLBP. The final 3 items concerned possible protective factors, namely positive affect (i.e., happiness and optimism) and pain acceptance. Each EMA item was formulated in a momentary timeframe (“at this moment”) to avoid recall bias. Items were presented in a randomized order at each moment.

At the start of each day, in addition to the momentary assessments, participants were asked once per day about their sleep quality the previous night. For this question, participants could indicate whether their sleep was *very bad, bad, neutral, good,* or *very good*. At the end of the day, participants were also asked whether they had experienced anything that day that may have influenced their ratings. This question was an optional open-ended question that could be completed once daily. In addition, participants were asked once per day about their medication and alcohol use. Each set of EMA items took approximately 3 minutes to complete, and questions within each EMA measurement were presented in a random order.

### 2.3. Procedure

Interested participants were sent an information letter through email to allow them to decide whether they agreed to participate. In this email, an appointment was made to conduct a screening call. Based on the phone call, participants who met the inclusion criteria were asked to come to the university faculty or schedule an online meeting for a detailed explanation of the study and to sign the consent form. On arrival at the research laboratory (or the online meeting room), participants were briefed about the study. Participants signed the (digital) consent form if they agreed to participate.

Participants then completed the set of questionnaires stated in the *cross-sectional measures* section. On completion of the questionnaires, participants were asked to download a mobile application called Ethica Data (now called Avicenna Research, https://avicennaresearch.com/). They were then assigned a pseudo email and password to ensure pseudonymity in registering with Ethica. After registration, the researcher discussed each of the momentary assessment questions with the participants to ensure a clear understanding of the momentary items. Participants were “beeped” randomly 5 times per day for 2 weeks, with the first “beep” set between 08:00 am—11:00 am The time of the first beep was adjusted based on the participants' self-reported waking hours. The second “beep” occurred between 11:00 am and 02:00 pm, then again at 02:00 pm—05:00 pm, 05:00 pm—08:00 pm, and the last “beep” between 08:00 pm—11:00 pm The final beep was adjusted according to the participants' sleeping schedule to avoid prompts during sleep time. Participants could respond to the EMA items within 30 minutes. If no responses were received within 30 minutes after the beep, then participants were sent a reminder notification within the designated time block.

At the end of the 2 weeks, participants were sent an exit questionnaire that included questions about their experience filling out the momentary assessments, what they thought about the study, and whether they had any suggestions for improvement.

### 2.4. Statistical analysis

#### 2.4.1. Data preparation

Data were prepared and analyzed using R version 2024.04.02 (network analyses) and IBM SPSS Statistics version 29.0 (descriptive analyses). Descriptive statistics for baseline variables such as age, sex, education level, and work status, were calculated and summarized in Table 1. To explore the data, we estimated both group and individual networks (see *Network Analysis* section for more details) and selected 6 main variables to explore, namely: pain, pain expectancies, pain avoidance, fear, attention and negative affect. These variables were selected as they have been shown to contribute to the maintenance of pain.^32^ High statistical power in network analyses often requires a large set of datapoints or fewer nodes to detect relationships (i.e., edges) between variables.^35^ Thus, to achieve higher statistical power by reducing the number of nodes in the network,^35^ variables which were measured using 2 related items and showed strong intercorrelations (all *p* < 0.001, *r* > 0.60) were combined into 1 node by calculating their mean score. These variables concern pain expectancies (i.e., stimulus and outcome expectancy questions), pain avoidance (i.e., pain avoidance and back harm avoidance), fear (i.e., emotional fear and worry), and negative affect (i.e., sadness and stress).

**Table 1:** Demographic characteristics.

Demographic factor	Value
Age	
M (SD)	46 (14.0)
Range	22-64
Sex (% female/male/other)	80/20/0
Education level (%)	
Secondary education	20
Tertiary education	80
Work status (%)	
Full time	23.3
Part time	36.7
Other	40
ODI	
M (SD)	27 (12.1)
Range	6-50
DASS-21	
Depression	
M (SD)	16.9 (12.5)
Range	0-22
Anxiety	
M (SD)	17.7 (13.1)
Range	0-24
Stress	
M (SD)	12.2 (10.0)
Range	0-28

ODI, Oswestry Disability Index (scale: 0-100 with higher numbers indicating higher disability); DASS-21, Depression, Anxiety, and Stress Scale (scale: 0-42 for each subscale, with higher numbers indicating higher depression, anxiety, and/or stress).

#### 2.4.2. Network analysis

##### 2.4.2.1. Assumption checks

As the data contained time series data, vector autoregressive (VAR) network models were fitted to explore the relationship between cognitive-affective and behavioral variables. To estimate a VAR model, a series of assumptions needed to be met namely: equidistant measures (meaning the time between each “beep” is proportional to one another), stationarity (meaning that the distribution of data does not change across time), and multivariate normality. As timing of the momentary assessments were randomized, and assessments were not measured overnight during sleep times, the timing of the assessments violated the assumption of equidistant measures. To address the overnight measurements, only the relations during the day (i.e., between the first, second, third, and fourth observations) were estimated. To check whether the data met the assumption of stationarity, linear regression models were fitted for all the variables to check for linear trends. Linear trends were detected in the variables *Pain*, *Attention*, and *Negative Affect*, indicating that these variables did not fit the assumption of stationarity; therefore, these variables were subsequently detrended and network analyses were repeated with detrended data. Finally, Kolmogorov–Smirnov tests were computed to check for normality which showed significant results for all variables (*p* < 0.001), indicating that all variables were not normally distributed. Given that there is no standardized method to address violations to normality, the data were analyzed in raw, imputed, and detrended format. Studies have also shown that violations to the assumption of equidistant measures and normality have no strong effect on the outcome of multivariate autoregressive models.^28,33,38^

##### 2.4.2.2. Network estimation

Networks consist of 2 main components, namely nodes (representing factors/variables) and edges (representing relations between the nodes) which can be depicted by arrows (in a directed network) or straight lines (in an undirected network). We first explored the group level data by estimating a multilevel vector autoregressive (mlVAR) model. This was performed using the R package *mlVAR*^14^ in which we estimated 2 types of time-series networks, namely, a temporal network and a contemporaneous network. The temporal network estimates a directed network that calculates whether one node predicts another node or itself (also known as autocorrelations) from one timepoint to the next timepoint (lag-1 relations), whereas the contemporaneous network estimates an undirected network that calculates how different nodes are connected to one another at one and the same given timepoint while controlling for all other nodes and all other timepoints (i.e., partial correlations). Contemporaneous networks may also contain an indication of VAR model misfit as indicated through the number and strength of edges.^16^ The *mlVAR* package uses listwise deletion by default to handle missing data, that is, it estimates a model based on the available data. However, we also used multiple imputations to handle missing data using the *mice* package in R^7^ to estimate the network with the imputed data set as an additional sensitivity analysis. To do so, we created 5 different imputed data sets,^7^ estimated separate mlVAR networks from those 5 imputed data sets, and pooled the networks by averaging the edge weights from those data sets into one imputed temporal and contemporaneous network.

We also assessed the individual time-series networks of all 30 participants separately by estimating a graphical vector autoregressive (gVAR) model. This was performed using the package *graphicalVAR* in R.^13^ Following the group level networks, we also estimated both temporal and contemporaneous networks for each individual. In case there were no variations in a variable within a participant, the gVAR network was estimated without this variable as a predictor (i.e., for participant 8, 15, 23, 24, and 30). Both mlVAR and gVAR models were plotted using the *qgraph* package in R.^15^ Residual analyses for each variable and each participant were computed to assess indications of model misfit in which residuals that are stable across time indicate good model fit.^23^ This can be obtained as an output through the *mlVAR* package version 0.5.4. For all networks, stronger relations are depicted by thicker edges. Blue edges indicate positive relations whereas red edges indicate negative relations.

## 3. Results

### 3.1. Participant characteristics

A total of 35 participants were included in the study. Of the 35 participants, 30 completed the ecological momentary assessments with less than 30% of missing data. As reported in the preregistration, those with more than 30% missing data were excluded from the main analyses. Thus, the final sample consisted of 30 participants, as planned, with a total of 7.5% missing data. Of 30 participants, 12 did not receive a diagnosis of primary CLBP made by a physician, but met the inclusion criteria according to the ICD-11 which are: pain localized between the costal margin and the inferior gluteal folds, lasting at least 3 months in duration, and characterized by emotional distress or functional disability.^26^ Participants reported having symptoms of chronic pain starting from 2 to 44 years ago but received an official diagnosis only between 1 and 14 years ago. Other demographic factors are summarized in Table 1. Most of the participants reported that they currently are or have had interventions to manage their pain in the past 3 months including psychological interventions (26.7%), physical therapy (83.3%), and other types of therapy (46.7%) such as acupuncture, osteopathy, and surgery. In addition, at least half of the final sample (53.3%) reported medication use to manage their pain in the past 3 months. Based on the results of the International Physical Activity questionnaire, 3 of 30 participants were sedentary, and the rest engaged in a mix of low, moderate, and high activity. Only 5 of the 30 participants performed intense physical activities on a regular basis.

The supplemental digital content (see File 2, http://links.lww.com/PAIN/C512) illustrates how the momentary factors assessed (i.e., pain, expectancies, avoidance, fear, attention, and negative affect) fluctuate over time. As shown in the figures, fluctuation patterns differ per individual and per factor. Pain had the most moment-to-moment variability, while fear and attention seem to be the most stable across participants. The level of fear and negative affect was low overall across participants, while avoidance and expectancies were in the upper range. Descriptive statistics, including the mean, standard deviation, and minimum and maximum value for each participant can be found in the upper right corner of each line plot in the supplemental digital content (see File 2, http://links.lww.com/PAIN/C512).

### 3.2. Networks

#### 3.2.1. Group-level networks

**Figure 1.**
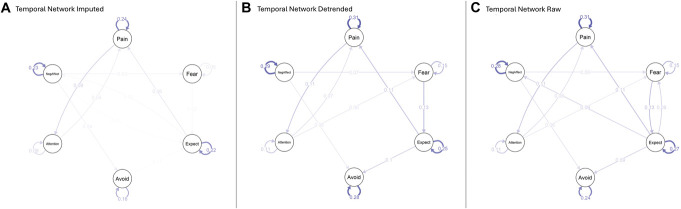
Group-level temporal networks with imputed data (A), detrended data (B), and raw data (C). Edges displayed in the network indicate significant relations (*p* < 0.05). Arrows indicate the direction of the relationship between nodes. Numbers within the edges indicate the strength of the relations, with thicker edges indicating stronger relations.

**Figure 2.**
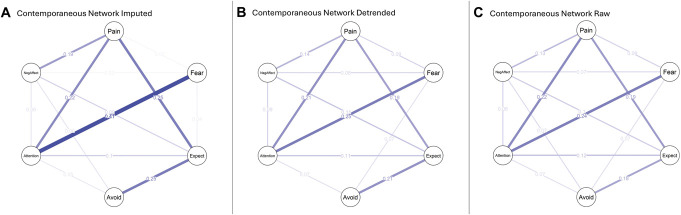
Group-level contemporaneous networks with imputed data (A), detrended data (B), and raw data (C). Edges displayed in the network indicate significant relationships between nodes (*p* < 0.05). Numbers within the edges indicate the strength of the relations, with thicker edges indicating stronger relations.

Figures 1 and 2 show the multilevel VAR networks of individuals with CLBP in this sample. The figures illustrate the group level networks using imputed, detrended, and raw data. All 3 networks show similar temporal and contemporaneous network structures which indicate that the connections between nodes are relatively stable. Figure 1A shows the temporal network of imputed data which represents the most stable network as the network was modeled from the most complete data obtained from averaging multiple data sets. Here, the strongest connections can be found between pain and attention, meaning that, on average, pain at one moment predicted attention to pain at the next moment. Similar relations can also be found between expectancy and pain. Interestingly, whereas avoidance weakly predicted expectancies in the imputed temporal network (Fig. 1A), the opposite direction was found in both the detrended and raw temporal networks (Figs. 1B and 1C). In all 3 networks, expectancy was a stable predictor of pain, pain was a stable predictor of attention, attention was a stable predictor of pain, negative affect was a stable predictor of avoidance and fear, and fear was a stable predictor of expectancy at the next moment.

In the imputed contemporaneous network, significant connections can be found between all nodes except between pain and avoidance, and avoidance and fear (Fig. 2A). In this network, the strongest connections were found between attention and fear, attention and pain, pain and expectancy, and expectancy and avoidance, and negative affect and pain. This was also reflected in the detrended and raw contemporaneous networks (Figs. 2B and 2C). Interestingly, all other contemporaneous connections (e.g., between pain and fear, and between negative affect and attention) show weaker edges in the imputed network compared with the detrended and raw contemporaneous networks. Overall, the strong edges in the contemporaneous networks indicate model misfit, which suggests that there may be individual differences in the individual level networks.^16^

#### 3.2.2. Individual networks

The supplemental digital content (see File 3, http://links.lww.com/PAIN/C512) shows the graphical VAR models of all 30 participants. All edges displayed indicate significant relations between nodes (*p* < 0.05). Both temporal and contemporaneous networks show high variability between participants. Networks ranged from highly dense networks (e.g., contemporaneous network of participant 12) to highly sparse networks (e.g., temporal network of participant 18) which may be due to the variability in time series data. It should also be noted that the graphical VAR model that we used to analyze the data had stringent requirements to detect an edge to lower the rate of false positives.^17^ Therefore, the edges that can be seen can be interpreted with higher confidence. The supplemental digital content (see File 4, http://links.lww.com/PAIN/C512) shows the residual plots for each variable and each participant across time. Owing to missing data, residuals were computed based on the smallest possible number of observations that can be predicted for each participant. These plots show that the residuals are relatively stable across time demonstrating no apparent patterns for model misfit. The mean, standard deviation, and minimum and maximum values for the residuals can be found in the upper right corner of the residual plots for each participant in the supplemental digital content (see File 4, http://links.lww.com/PAIN/C512). For the current article, we will discuss 3 individual networks with a mix of sparse and dense networks to illustrate the network variation across participants.

**Figure 3.**
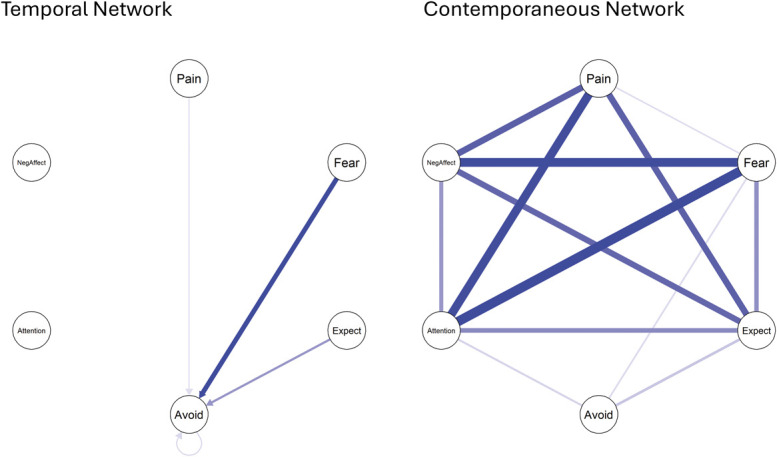
Temporal and contemporaneous network of participant 12.

**Figure 4.**
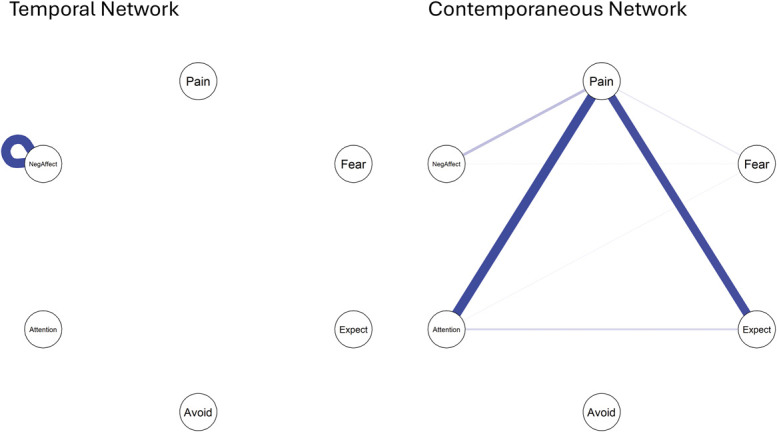
Temporal and contemporaneous network of participant 5.

**Figure 5.**
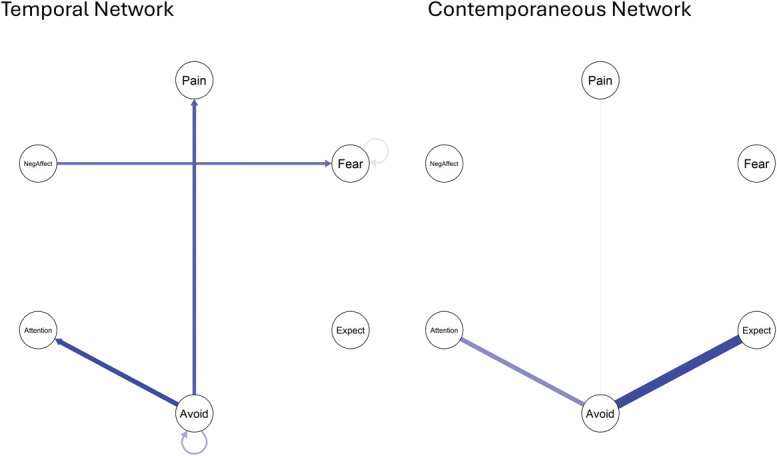
Temporal and contemporaneous network of participant 7.

Figure 3 shows the temporal and contemporaneous network of participant 12. The strongest temporal relations can be found between fear and avoidance, whereas strong contemporaneous relations can be found among most variables. This was the only contemporaneous network on an individual level with a similar structure to the group-level network. Figure 4 shows the temporal and contemporaneous network of participant 5. This participant had a sparse temporal network where the only edge can be found in the autocorrelation of negative affect. The strongest contemporaneous connection for this participant can be found between attention, pain, and expectancies. Figure 5 shows the temporal and contemporaneous network of participant 7. For this participant, more connections were found in their temporal network, as opposed to their contemporaneous network—opposite of the group-level network. Strong temporal connections can be found between negative affect and fear, avoidance and pain, and avoidance and attention, while the strongest contemporaneous connections are only between expectancy, avoidance, and attention. Overall, these networks demonstrate the individual variations of pain mechanisms that play a role in individuals' daily life.

## 4. Discussion

This study explored the relationship between pain, expectancy, avoidance, fear, attention to pain, and negative affect. We explored this relationship by estimating a temporal network to assess the relationship between pain and cognitive-affective and behavioral factors across time, as well as a contemporaneous network to assess relations in the same time window, on both a group and individual level. The findings from these networks demonstrate how different cognitive-affective and behavioral factors reinforce each other momentarily and across time. However, the relations between different cognitive-affective and behavioral factors vastly differ across individuals.

On a group level, across all temporal and contemporaneous networks, the (predictive) relationship between expectancy and pain, and pain and attention seem to be the most consistent. This supports the notion that expectancy of pain influences pain perception,^1,49^ and that pain draws attention,^21,43,44^ while adding to the literature that attention at one moment may play an important role in pain persistence and can also predict pain perception. It should also be considered that the relationship between pain and attention may be influenced by the attention to pain that is needed to report the level of pain. Furthermore, the contemporaneous network shows that fear and attention are highly correlated within the same timeframe—a finding that is in line with other non-network momentary assessment studies (e.g., Ref. 10, 51). However, it should be noted that unlike previous studies, the directionality of the relationship between fear and attention cannot be inferred from the current data. Therefore, it is unclear whether attention draws fear, whether fear draws attention at the same moment, or whether there is a reciprocal relationship between the 2 variables. In addition, it is important to highlight that the level of fear in this sample was relatively low thus the fear-attention relationship might not be generalizable to high levels of fear.

Interestingly, there was no direct relationship between pain and avoidance in both group-level temporal and contemporaneous networks, and only a weak relationship between fear and avoidance in the contemporaneous network. This is contrary to the fear-avoidance model of pain which proposes that avoidance is one of the key mechanisms in the maintenance of pain.^63,65^ However, it should be noted that the fear-avoidance relationship can be found in several individual networks (e.g., contemporaneous network of participants 4, 18, and 27, and temporal network of participants 12, 14, and 16). As edges detected in individual networks are often the strongest connections, this may indicate that the fear-avoidance mechanism is only at play in some individuals but cannot be generalized to all individuals with chronic pain. In addition, the absence of a negative relationship between avoidance and pain could indicate that the avoided daily activity may not have been the source of pain in the first place, demonstrating that avoidance may not be an effective strategy in reducing chronic pain. If the avoidance strategy was effective in reducing pain, a negative relationship would have been observed between avoidance and pain. This aligns with reports describing that the occurrence of pain is difficult to predict for individuals with chronic pain thus making it difficult to avoid.^37,61^ A more effective strategy may be to draw attention away from pain by (re)engaging in valued life activities or to challenge dysfunctional pain expectations.

When looking at individual networks, there is a large variability in how different cognitive-affective and behavioral factors relate to one another. This may have been influenced by the weaker statistical power or variation on an individual level which may have led the model to only detect the strongest edges. Thus, the same cognitive-affective and behavioral factors may have different relations on different participants. As exhibited by the difference in networks between participant 12 and participant 7, pain was the predictor of avoidance at the next moment in participant 12, whereas the opposite direction was found for participant 7. This demonstrates that pain affects each individual differently and that generalizing from group data to individual patterns is precarious.^66^ In addition, except for the contemporaneous network of participant 12, the group level networks were not reflected in any of the individual networks. This could be indicative of individualized treatment being a more optimal approach to manage chronic pain, especially given that treatments developed based on group-level data do not always work for all individuals.^54^ Some studies have already begun to use the network approach to individualize treatment for psychopathology,^31^ and the same could be applied for chronic pain treatment (e.g., Ref. 25). With the development of network-based interventions, it is worth noting the methodological challenges that may affect the network outcome, particularly with node selection (i.e., deciding which factors to assess), the timing of the measurements, and how items are measured. These measurement components can affect both the structure of the network and the interpretation of the network and should be thoroughly considered while developing the intervention.

Furthermore, considering that many studies investigating mechanisms underlying chronic pain are momentary, differences in the temporal and contemporaneous network structure highlight the need for more intensive longitudinal studies (e.g., using frequent momentary assessments) to better understand chronic pain. In addition, similarity and differences in network structures were found between the imputed, detrended, and raw networks. That being said, many relations remain consistent across all networks, namely between pain and attention, and expectancies and pain demonstrating the robustness of the relationship between these variables. This may indicate that these cognitive-affective factors may be suitable targets in an intervention, especially given that these factors are modifiable.^48,57^

Although this study highlights the value of investigating temporal relations across cognitive-affective and behavioral factors in chronic pain, some limitations need to be addressed. First, the current data violate the assumption of normality and stationarity required to estimate a VAR model. Yet, to date, there are no standardized guidelines on how to address these violations, and alternative methods to address these violations may not always be feasible.^5,39^ In addition, detrending was used to address the issue of nonstationarity. However, detrending is most often used in cases of linear trends. It is important to consider alternative methods to address nonstationarity for other trends such as using time-varying network models,^22,53^ differencing,^52^ and regime switching.^29^ Future studies could benefit from assessing longitudinal data using techniques that are more robust against violations of assumptions. Second, temporal and contemporaneous networks only depict correlations between variables, making it difficult to infer causal relations from the model. One method for future studies to assess causal relations may be to assess changes in a network after a triggered event and compare the network structure before and after the event. Another method is the invariant causal prediction method,^68^ which allows the assessment of causal relations between multiple variables. Having evidence to support causal relations can strengthen the current theoretical models that explain the relationship between different cognitive-affective and behavior factors in chronic pain. Third, the current sample may not represent the full spectrum of individuals with CLBP as most participants had mild levels of disability, were physically active, and have undergone treatment to manage their chronic pain. Those who had more severe symptoms may have been less inclined to participate in an intensive longitudinal study. Therefore, future studies could be conducted with a more heterogenous sample with varying symptom severity and various types of chronic pain (e.g., chronic widespread pain, or migraine) to increase generalizability. A different network structure in another chronic pain condition can already be seen in individuals with genital pain.^11^ In addition, it may be beneficial to investigate the relations between cognitive-affective and behavioral factors by incorporating different sources of data (e.g., physiological data). This has already been performed in a few studies^59,60^ and can potentially provide a more comprehensive picture of the mechanisms behind chronic pain.

### 4.1. Conclusion

This study was the first study to investigate the temporal network of cognitive-affective and behavioral factors in individuals with CLBP. The results suggest that various factors reinforce one another over time to maintain chronic pain. In addition, different cognitive-affective and behavioral factors are at play in different individuals, suggesting that a more individualized approach to chronic pain treatment is needed for better treatment outcomes. Nevertheless, these results demonstrate that data at both group and individual levels are needed to gain a comprehensive view of the factors related to CLBP and other chronic conditions. Using the network approach may help capture the complexity behind chronic pain mechanism and reveal more accurate targets for effective (individualized) treatment.

## Conflict of interest statement

The authors have no conflict of interest to declare.

## Supplemental digital content

Supplemental digital content associated with this article can be found online at http://links.lww.com/PAIN/C512.

## Supplementary Material
