# Supplementary material for: Cognitive-affective and behavioral pain mechanisms in individuals with chronic low back pain: a network analysis
Source: Pain. 2026 Jun 16;167(8):1958–67. doi: 10.1097/j.pain.0000000000004020 (PMC13382869; doi:10.1097/j.pain.0000000000004020)

SUPPLEMENTARY FILE 1

English Translation of Ecological Momentary Assessment Items

Variables	*Items*							
	Please indicate to what extent you agree with the following statements <u>*at this moment:*</u>							
		Not at all 0						*Extremely 6*
Pain Intensity	I have pain							
Fatigue	I have fatigue							
Expectancy- *(Outcome)*	I expect to feel pain in the next two hours							
		Not at all 0						*Extremely 6*
Expectancy - *(Stimulus)*	I expect that my pain will worsen when I engage in my daily activities							
Avoidance	I avoid activities that (I expect) will worsen my pain							
	I avoid activities that (I expect) will harm my back							
Fear	My pain makes me worry							
	My pain frightens me							
Attention	I am focusing on my pain							
Pain Acceptance	I can accept my pain							
Affect	I feel sad							
	I feel happy							
	I feel calm							
	I feel stressed							

Ecological Momentary Assessment Items in its Original Language (Dutch)

Variabele	*Item*							
	Geef aan in hoeverre je het eens bent met de volgende stellingen *op dit moment:*							
		Helemaal niet 0						*Extreem 6*
Pijnintensiteit	Heb ik pijn							
Vermoeidheid	Ben ik vermoeid							
Verwachting - *(Uitkomst)*	Verwacht ik pijn in de komende twee uur							
		Helemaal niet 0						*Extreem 6*
Verwachting - *(Stimulus)*	Verwacht ik dat mijn pijn erger zal worden als ik deelneem aan mijn dagelijkse activiteiten							
Vermijding	Vermijd ik activiteiten waarvan ik verwacht dat ze mijn pijn erger zullen maken							
	Vermijd ik activiteiten waarvan ik verwacht dat ze mijn rug schaden							
Angst voor de pijn	Maak ik mij zorgen over mijn pijn							
	Maakt mijn pijn mij bang							
Aandacht	Richt ik mijn aandacht op mijn pijn							
Pijnacceptatie	Kan ik mijn pijn accepteren							
Beïnvloeden	Voel ik mij verdrietig							
	Voel ik mij blij							
	Voel ik mij kalm							
	Voel ik mij gestrest							

SUPPLEMENTARY FILE 2

Momentary changes in level of pain for each participant

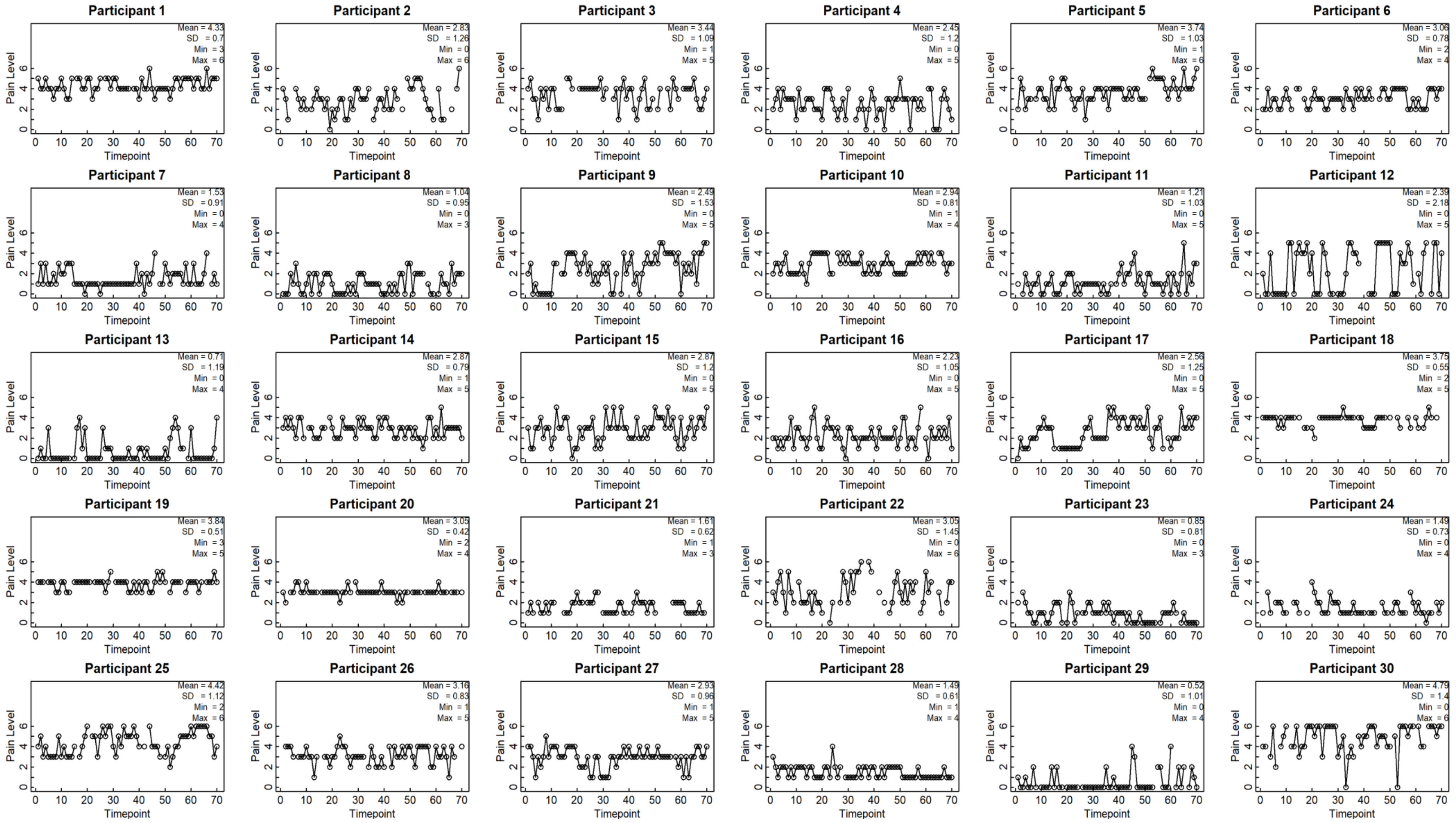

Momentary changes in level of expectancy per participant

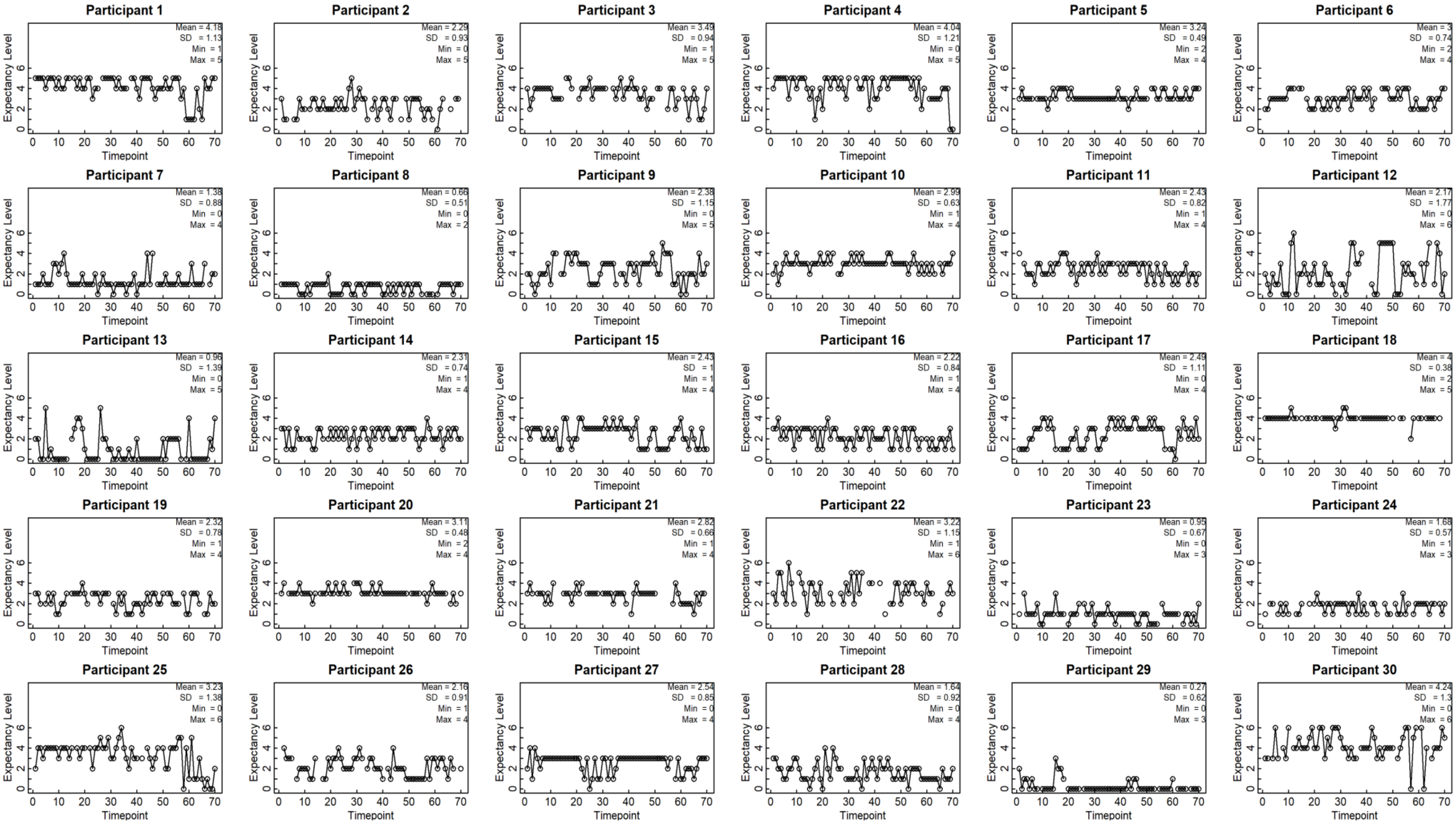

Momentary changes in level of avoidance per participant

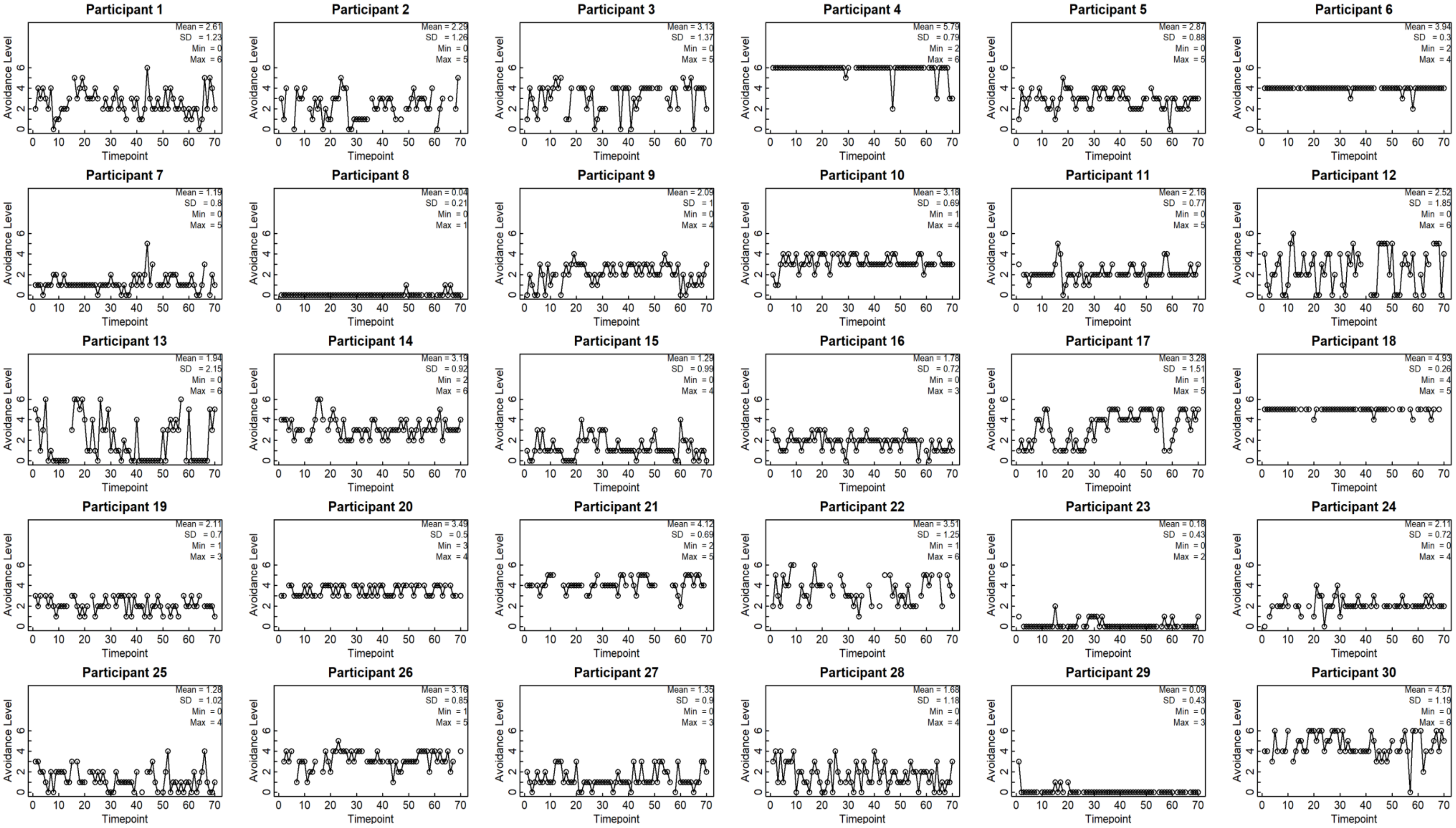

Momentary changes in level of fear per participant

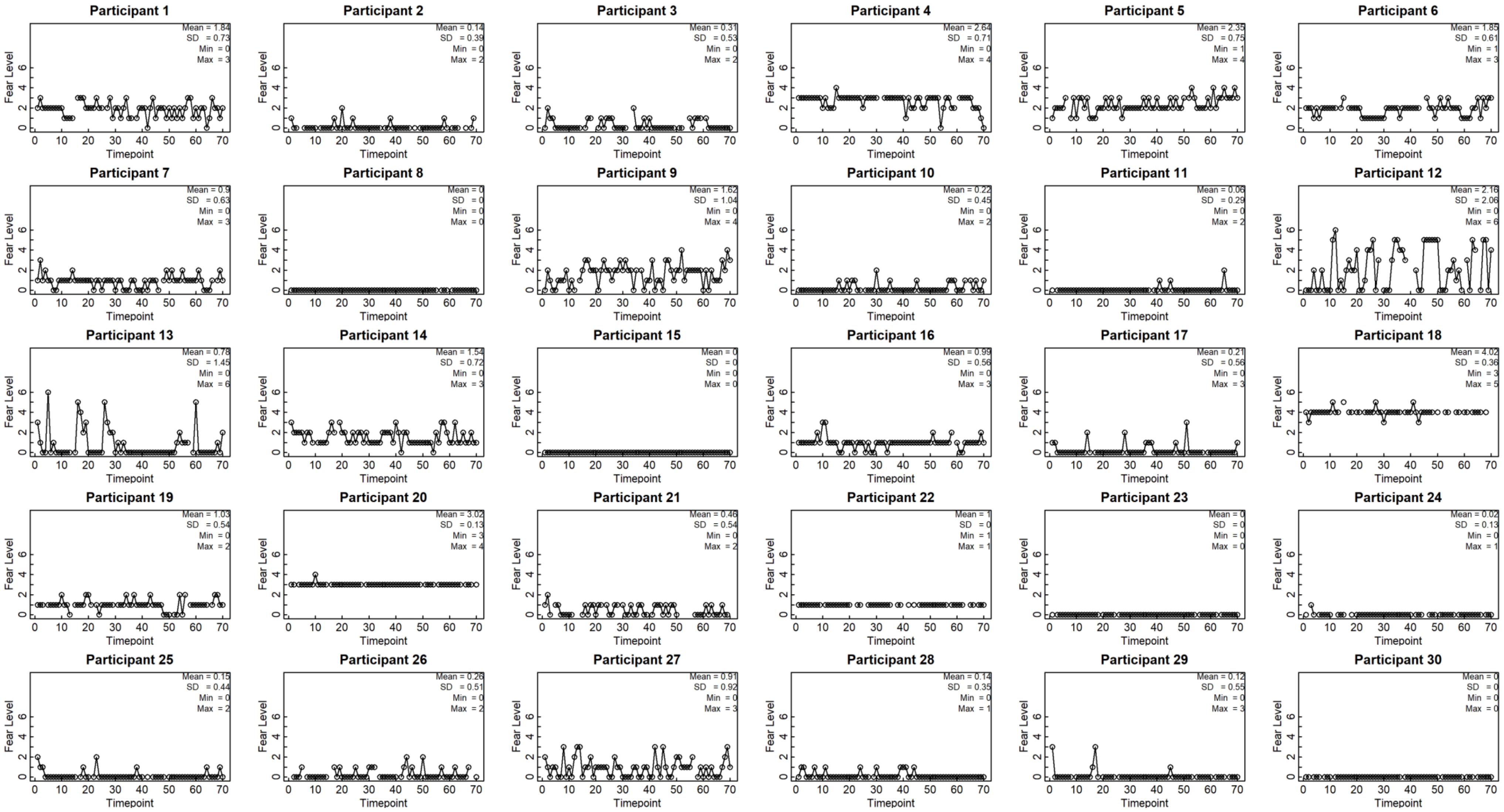

Supplementary File 2 – Cognitive-affective and behavioral pain mechanisms in individuals with chronic low back pain: a network analysis
by Nadinda, van Laarhoven, Waldorp, Vlaeyen, Peters, Evers

Momentary changes in level of attention to pain per participant

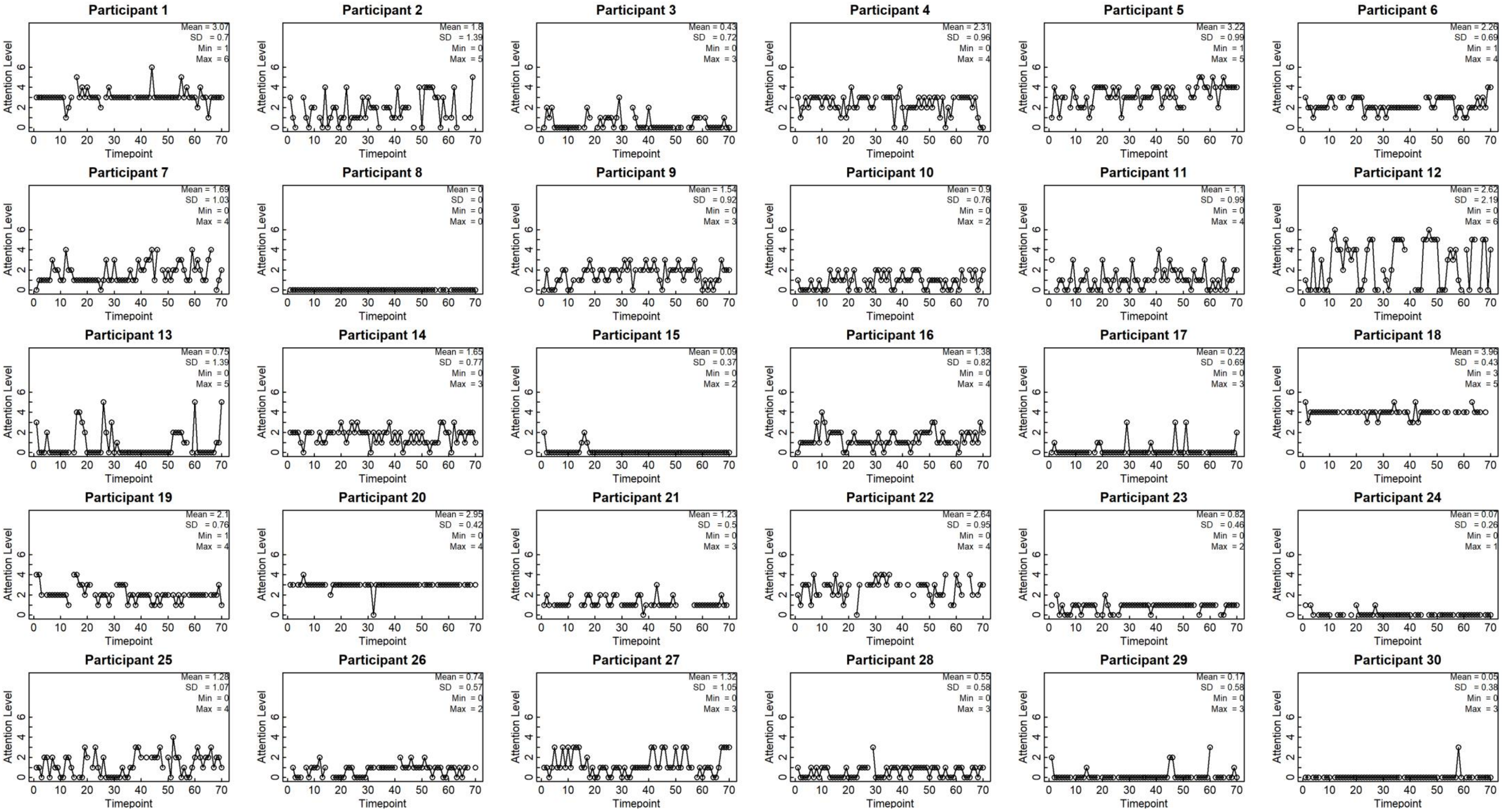

Momentary changes in level of negative affect per participant

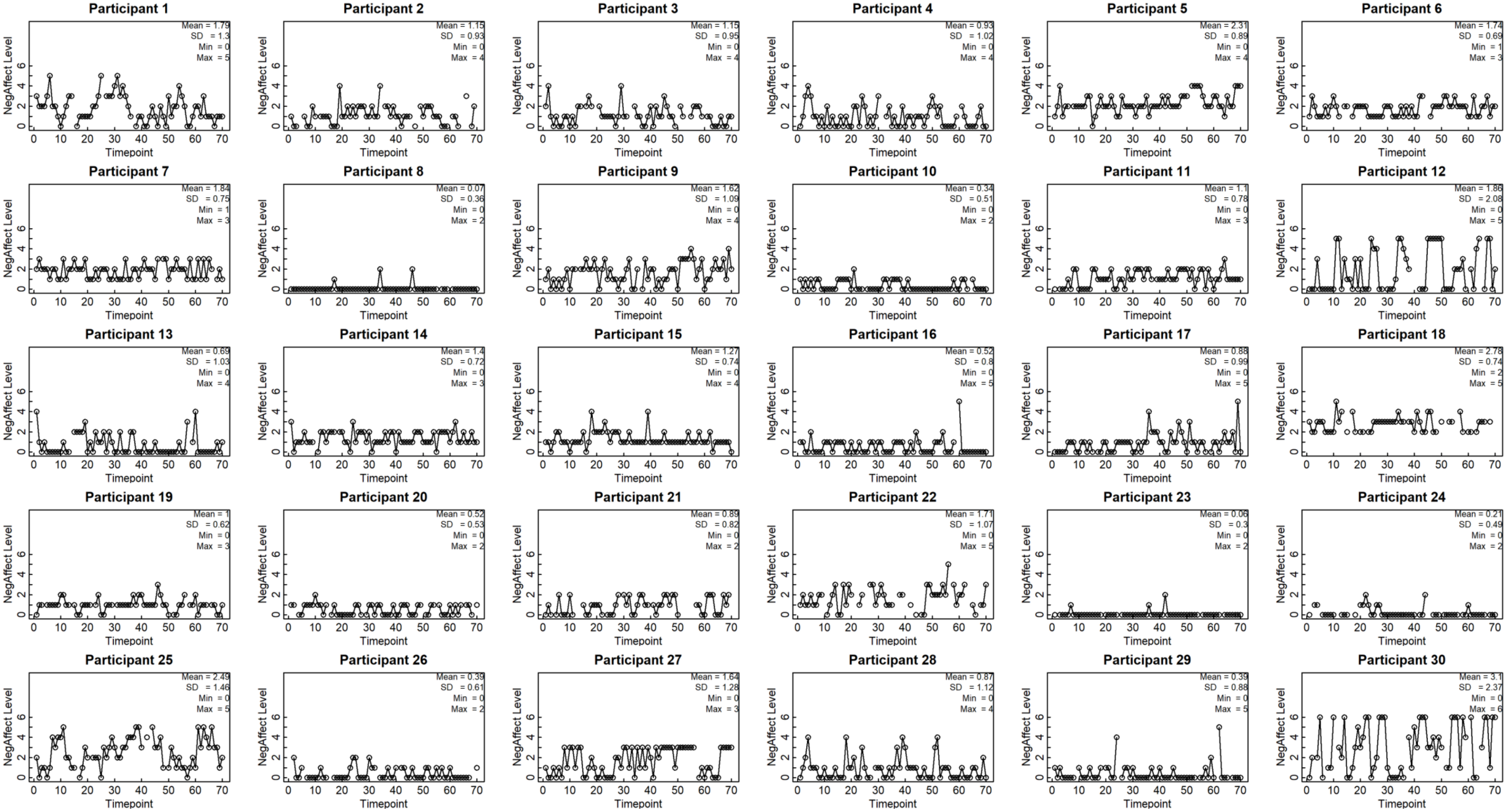

SUPPLEMENTARY FILE 3

Temporal network of each participant based on imputed data

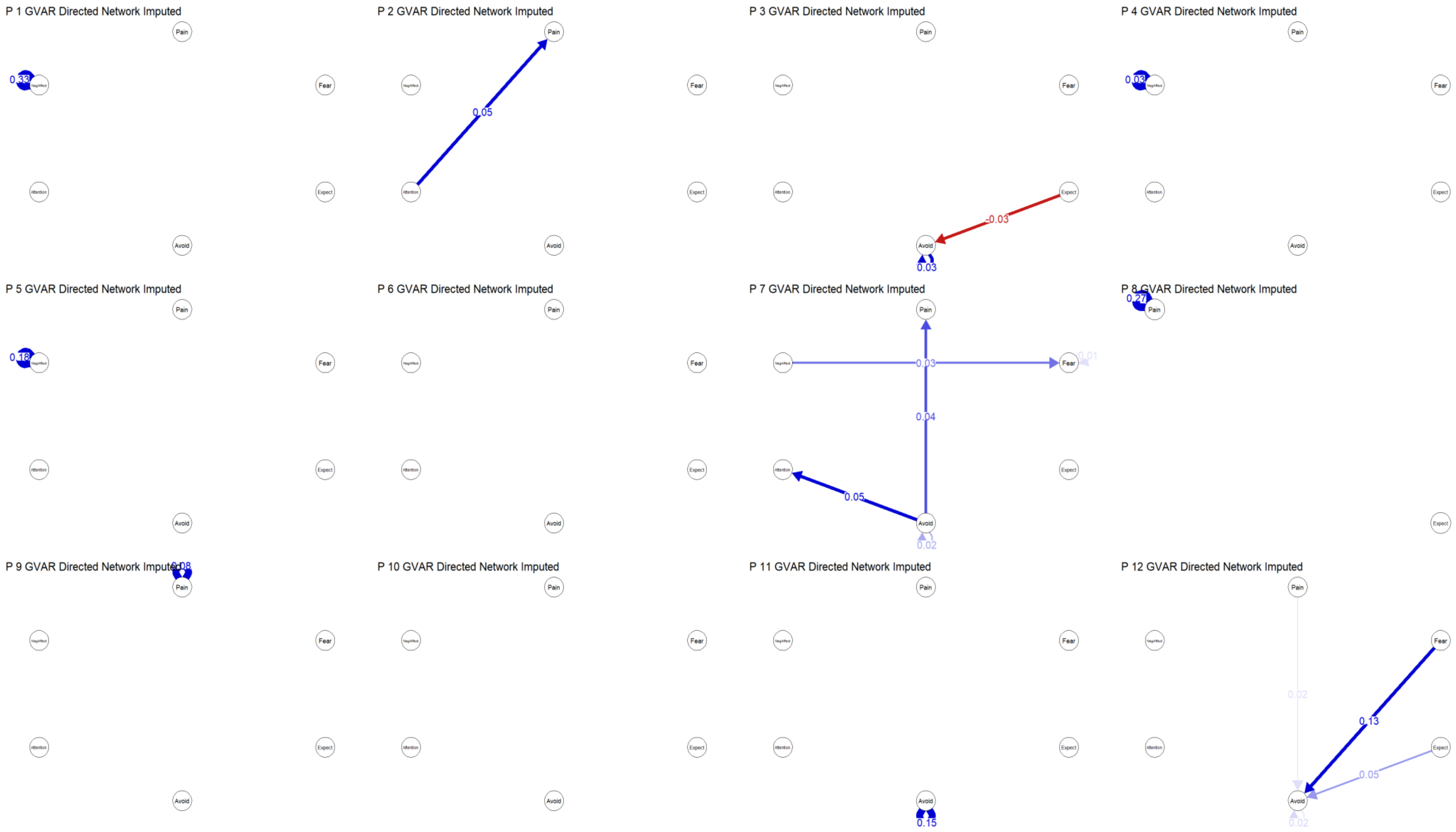

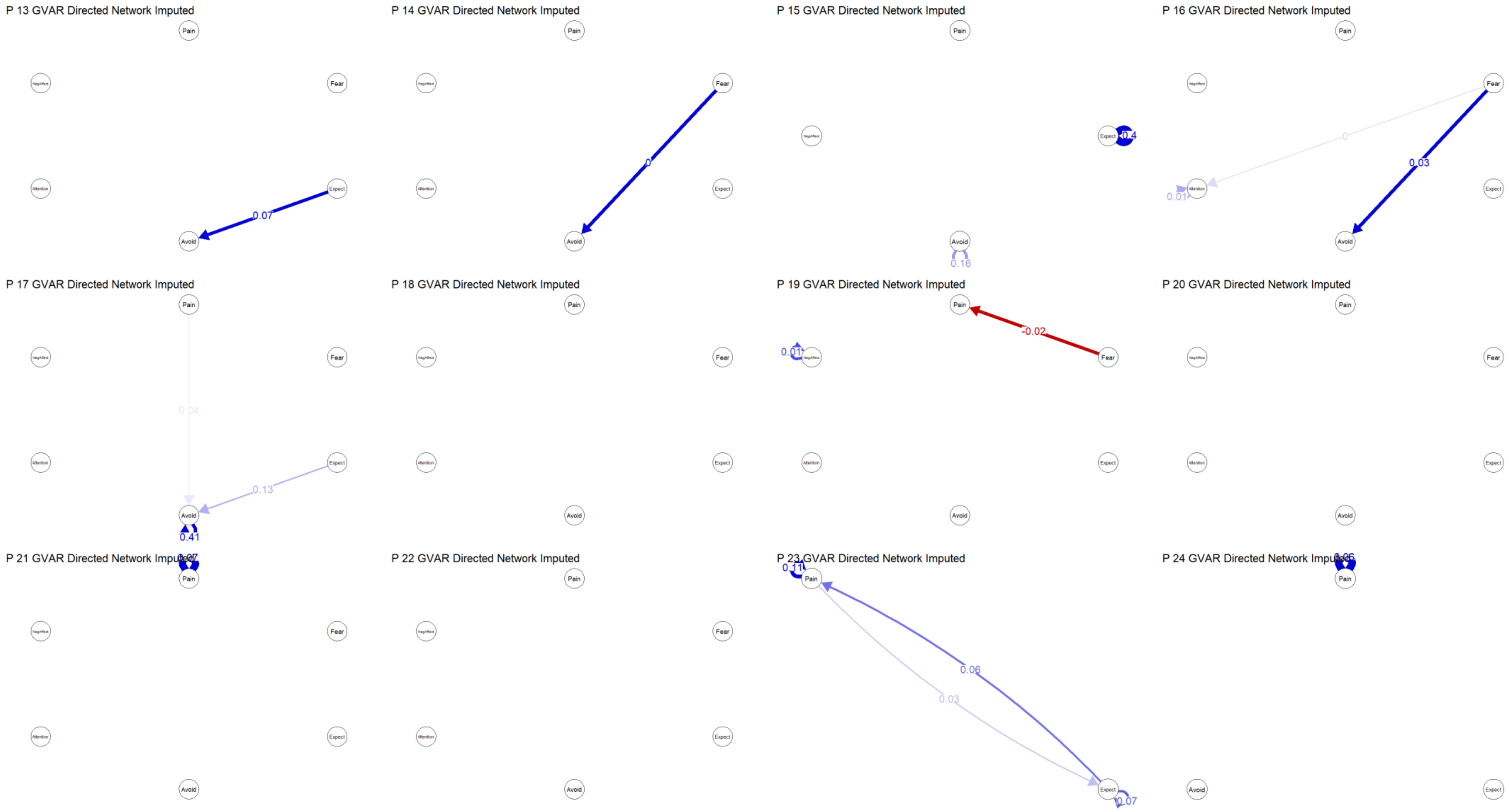

Supplementary File 3 – Cognitive-affective and behavioral pain mechanisms in individuals with chronic low back pain: a network analysis by Nadinda, van Laarhoven, Waldorp, Vlaeyen, Peters, Evers

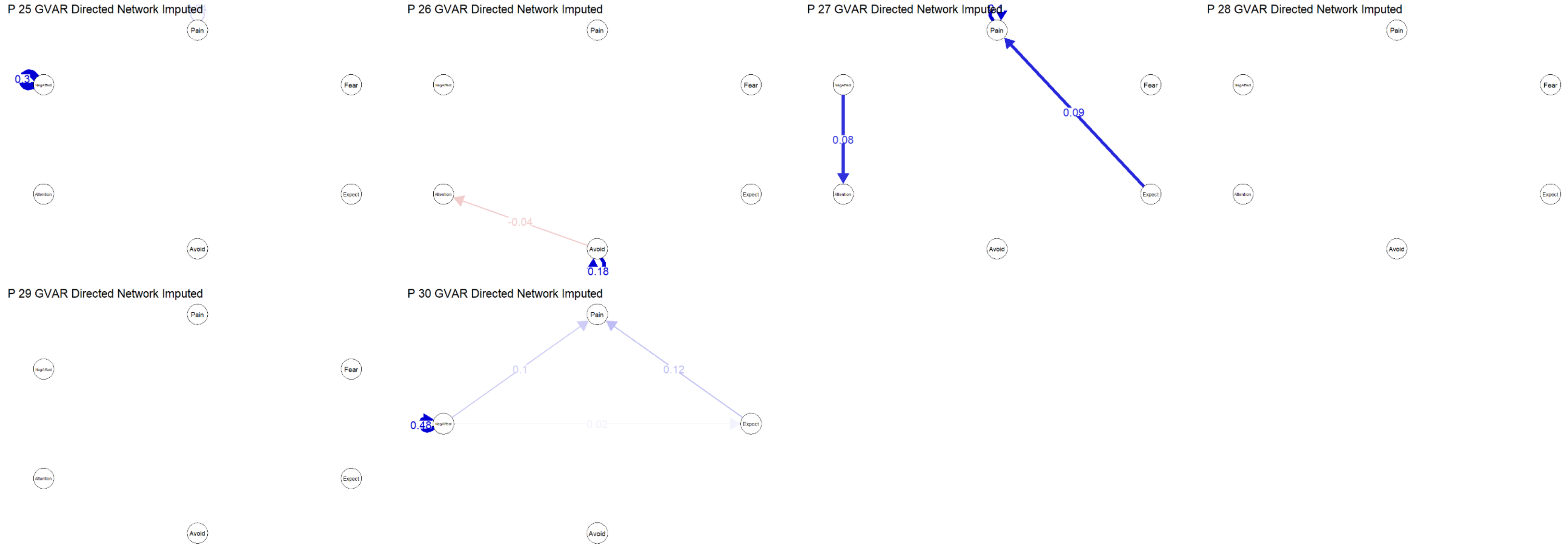

Supplementary File 3 – Cognitive-affective and behavioral pain mechanisms in individuals with chronic low back pain: a network analysis by Nadinda, van Laarhoven, Waldorp, Vlaeyen, Peters, Evers

Contemporaneous network of each participant based on imputed data

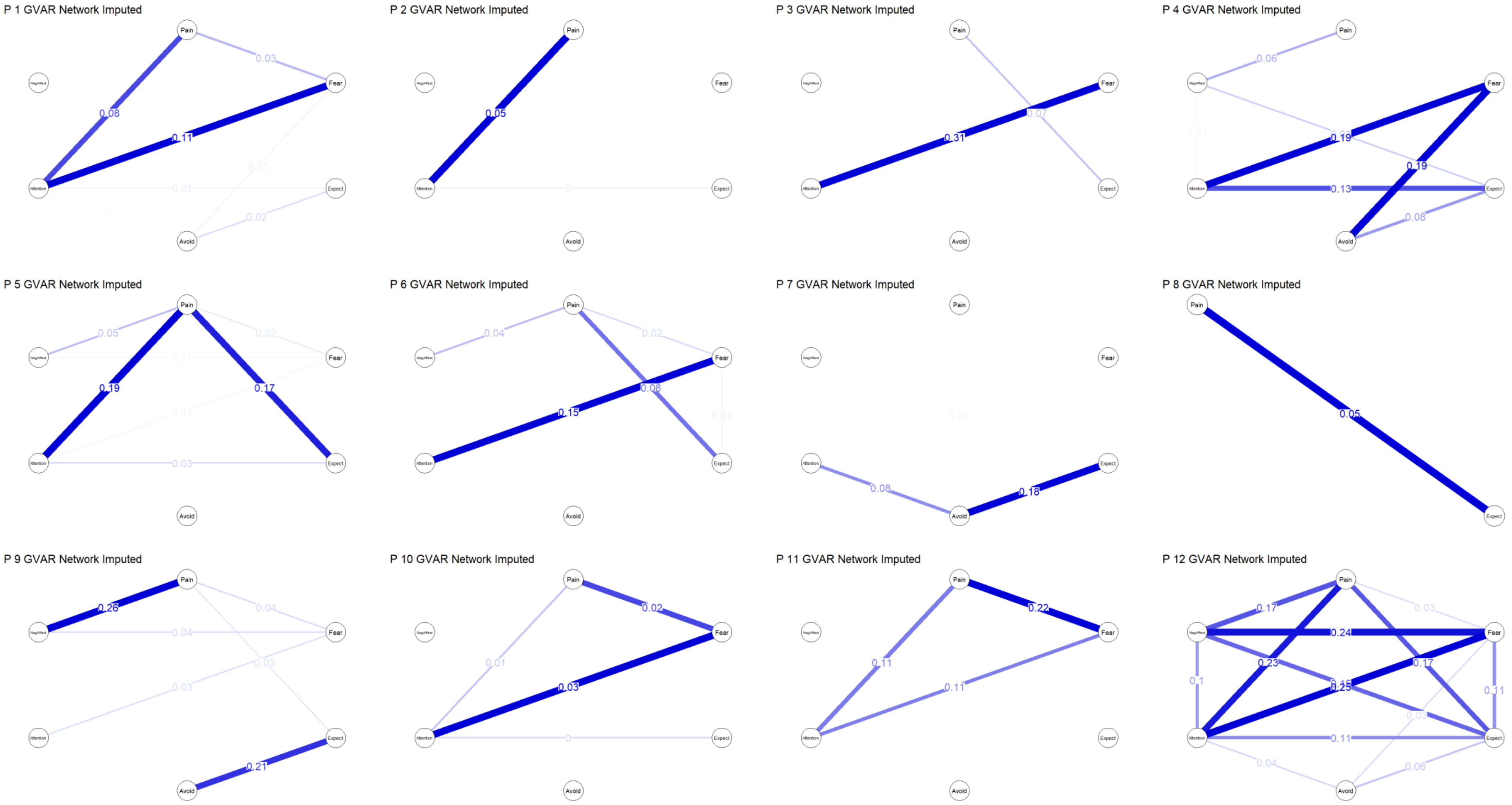

Supplementary File 3 – Cognitive-affective and behavioral pain mechanisms in individuals with chronic low back pain: a network analysis by Nadinda, van Laarhoven, Waldorp, Vlaeyen, Peters, Evers

P 13 GVAR Network Imputed

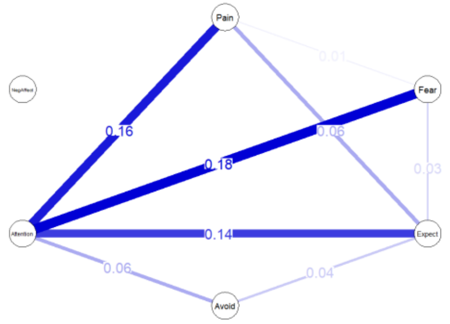

P 14 GVAR Network Imputed

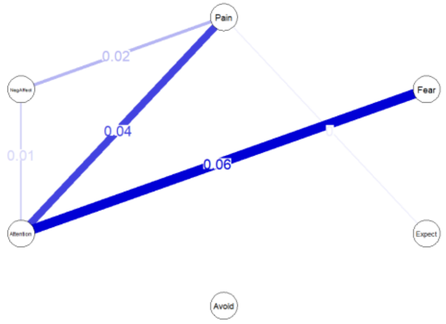

P 15 GVAR Network Imputed

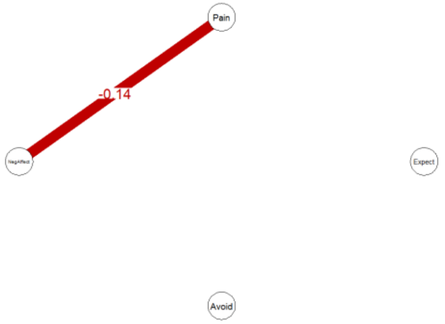

P 16 GVAR Network Imputed

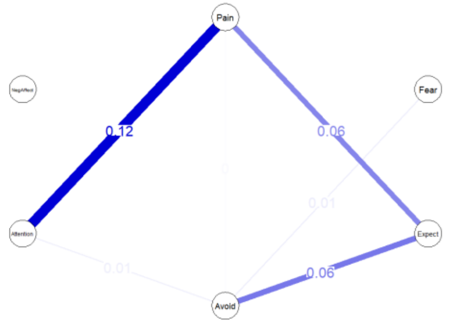

P 17 GVAR Network Imputed

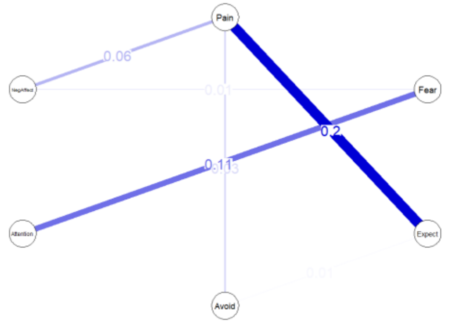

P 18 GVAR Network Imputed

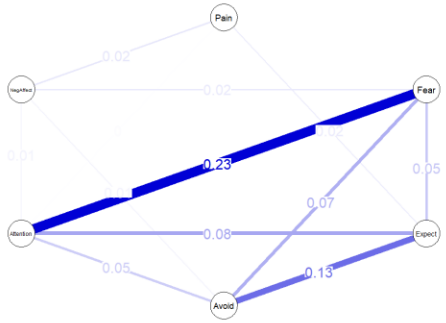

P 19 GVAR Network Imputed

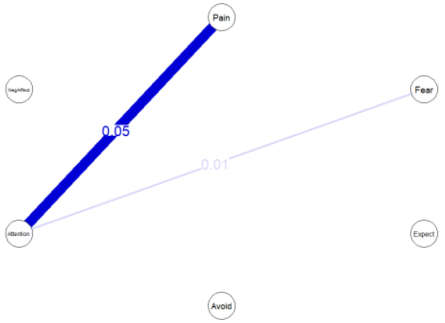

P 20 GVAR Network Imputed

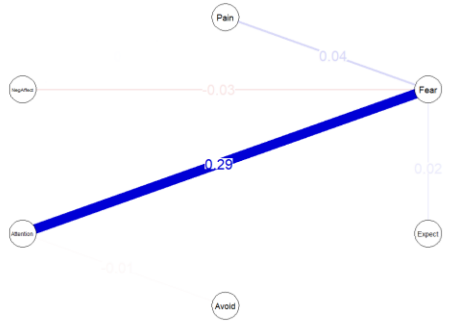

P 21 GVAR Network Imputed

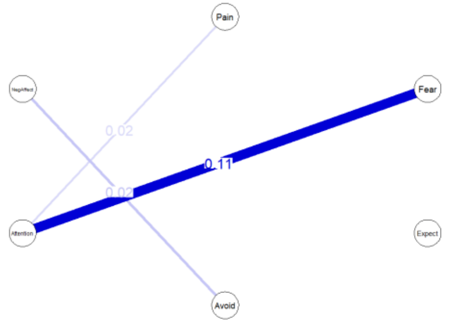

P 22 GVAR Network Imputed

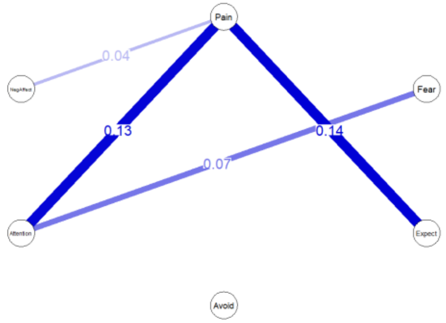

P 23 GVAR Network Imputed

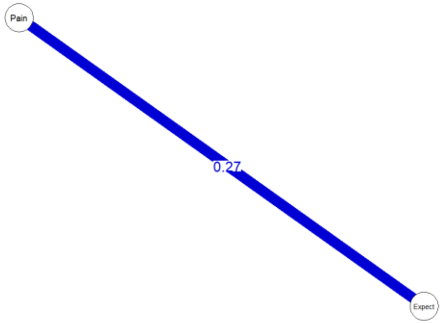

P 24 GVAR Network Imputed

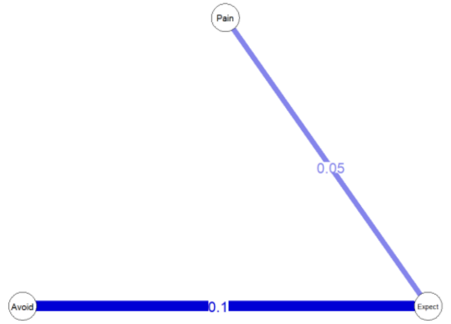

Supplementary File 3 – Cognitive-affective and behavioral pain mechanisms in individuals with chronic low back pain: a network analysis by Nadinda, van Laarhoven, Waldorp, Vlaeyen, Peters, Evers

P 25 GVAR Network Imputed

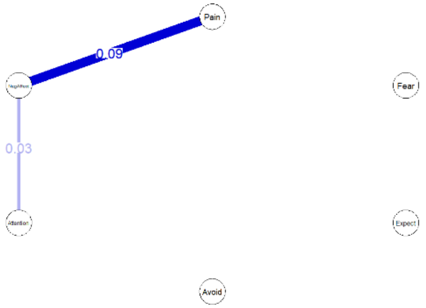

P 26 GVAR Network Imputed

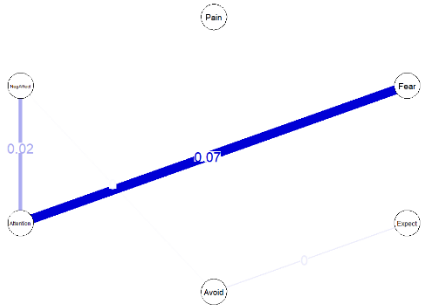

P 27 GVAR Network Imputed

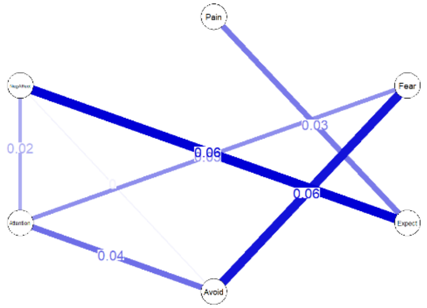

P 28 GVAR Network Imputed

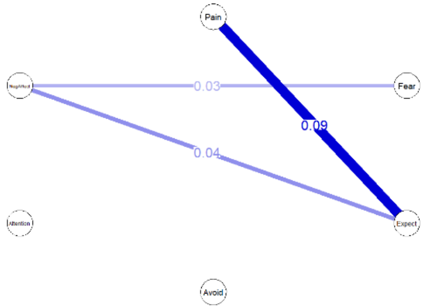

P 29 GVAR Network Imputed

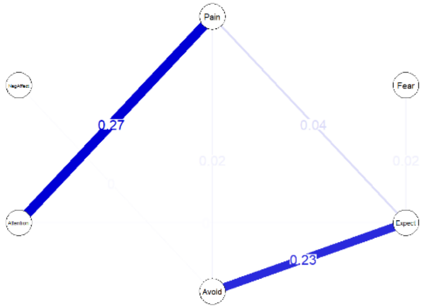

P 30 GVAR Network Imputed

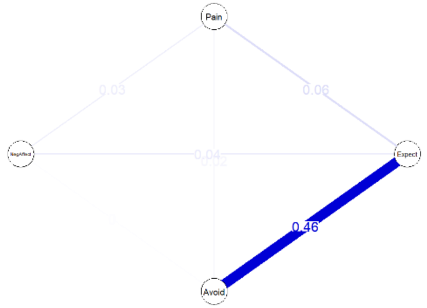

SUPPLEMENTARY FILE 4

Pain residuals across time for each participant

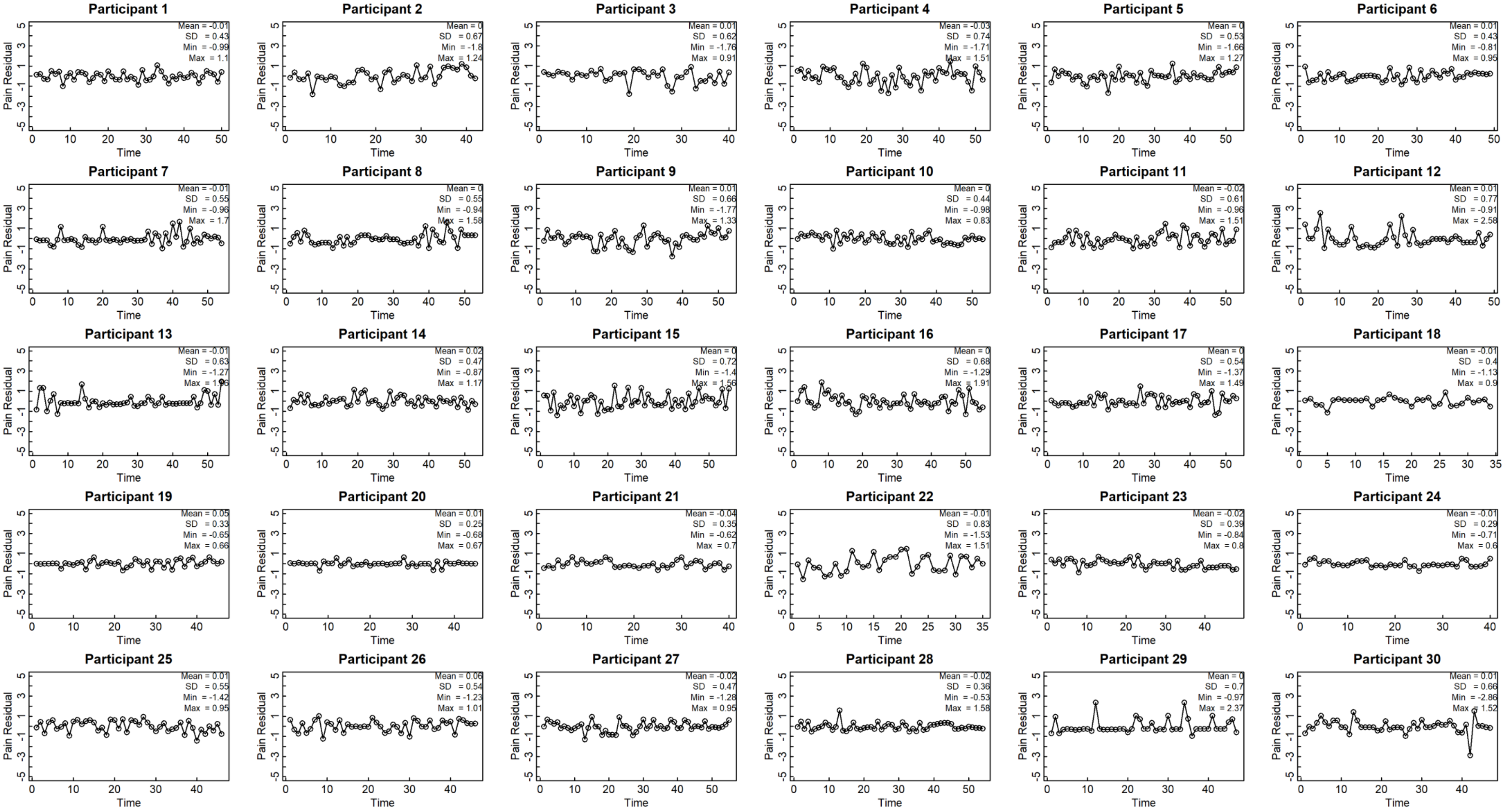

Expectancy residuals across time for each participant

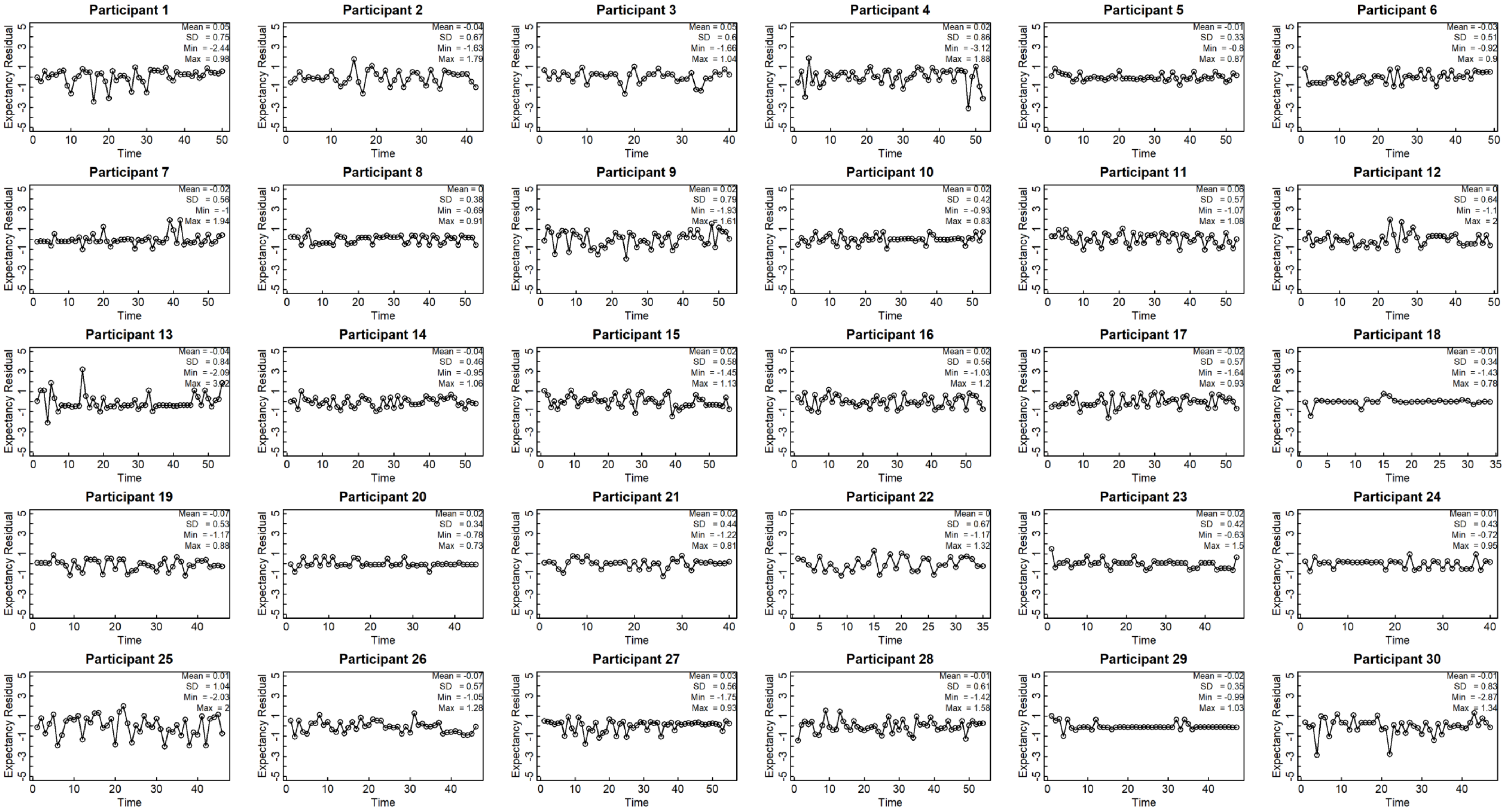

Avoidance residuals across time for each participant

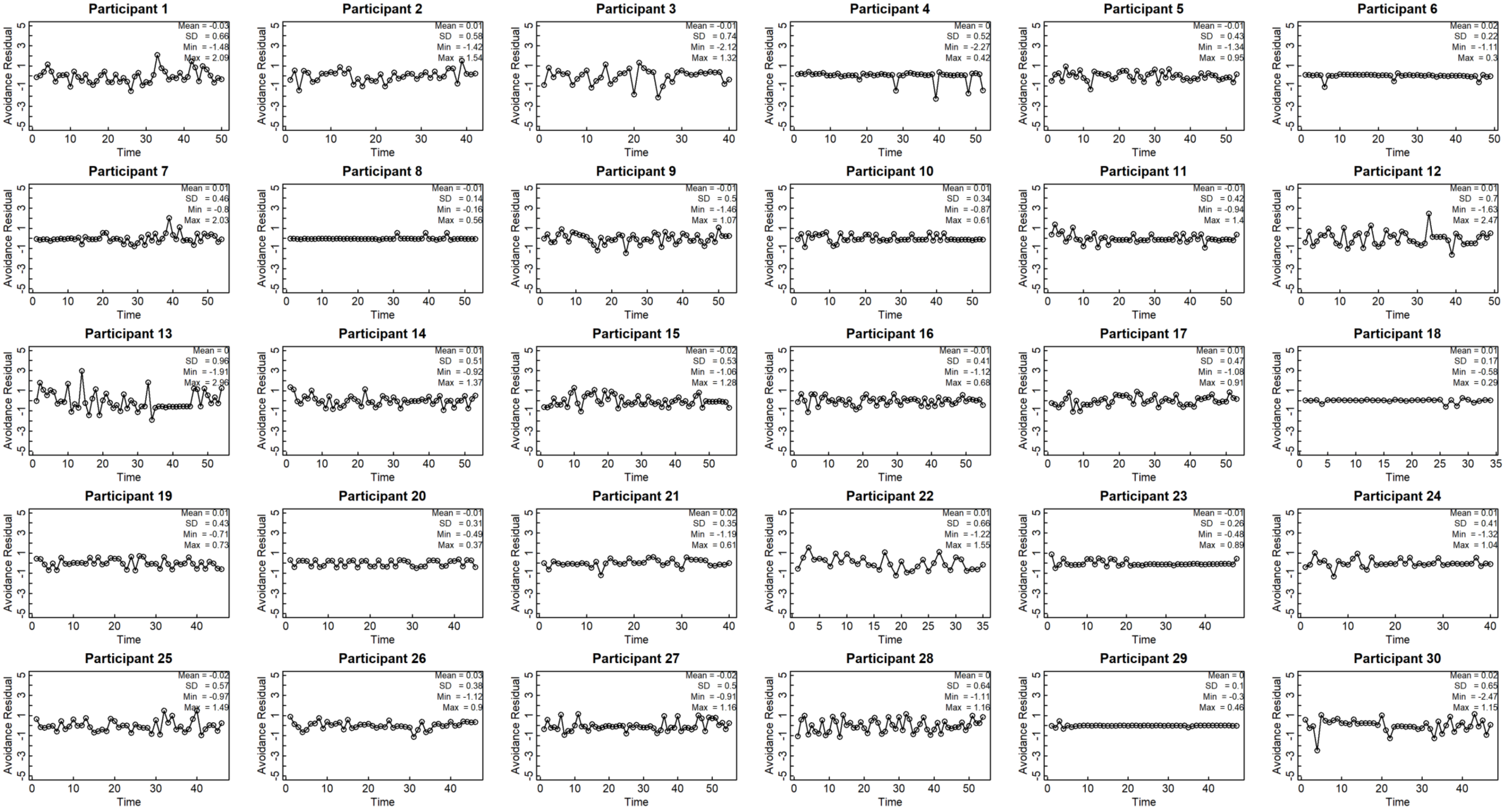

Fear residuals across time for each participant

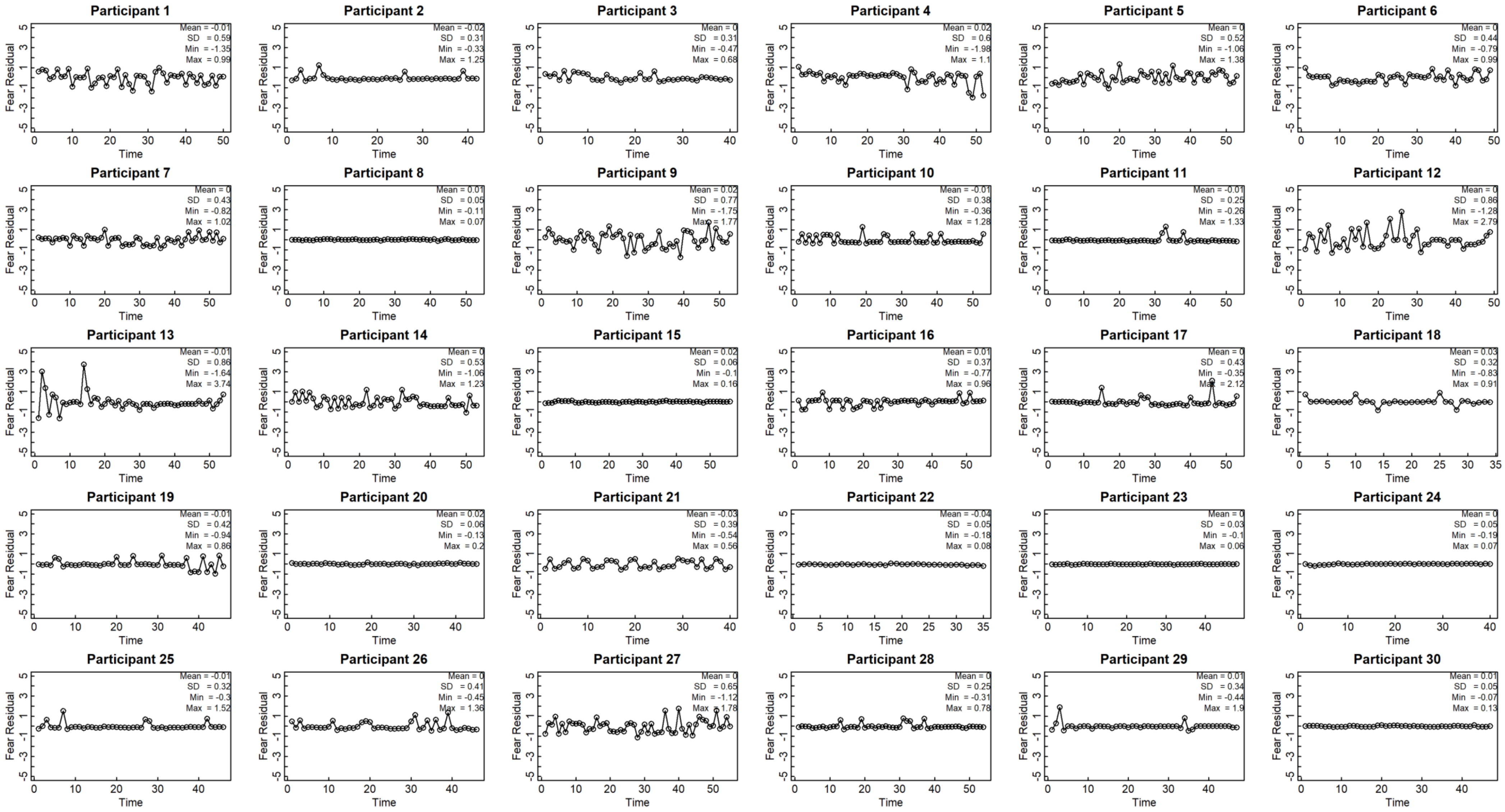

Attention residuals across time for each participant

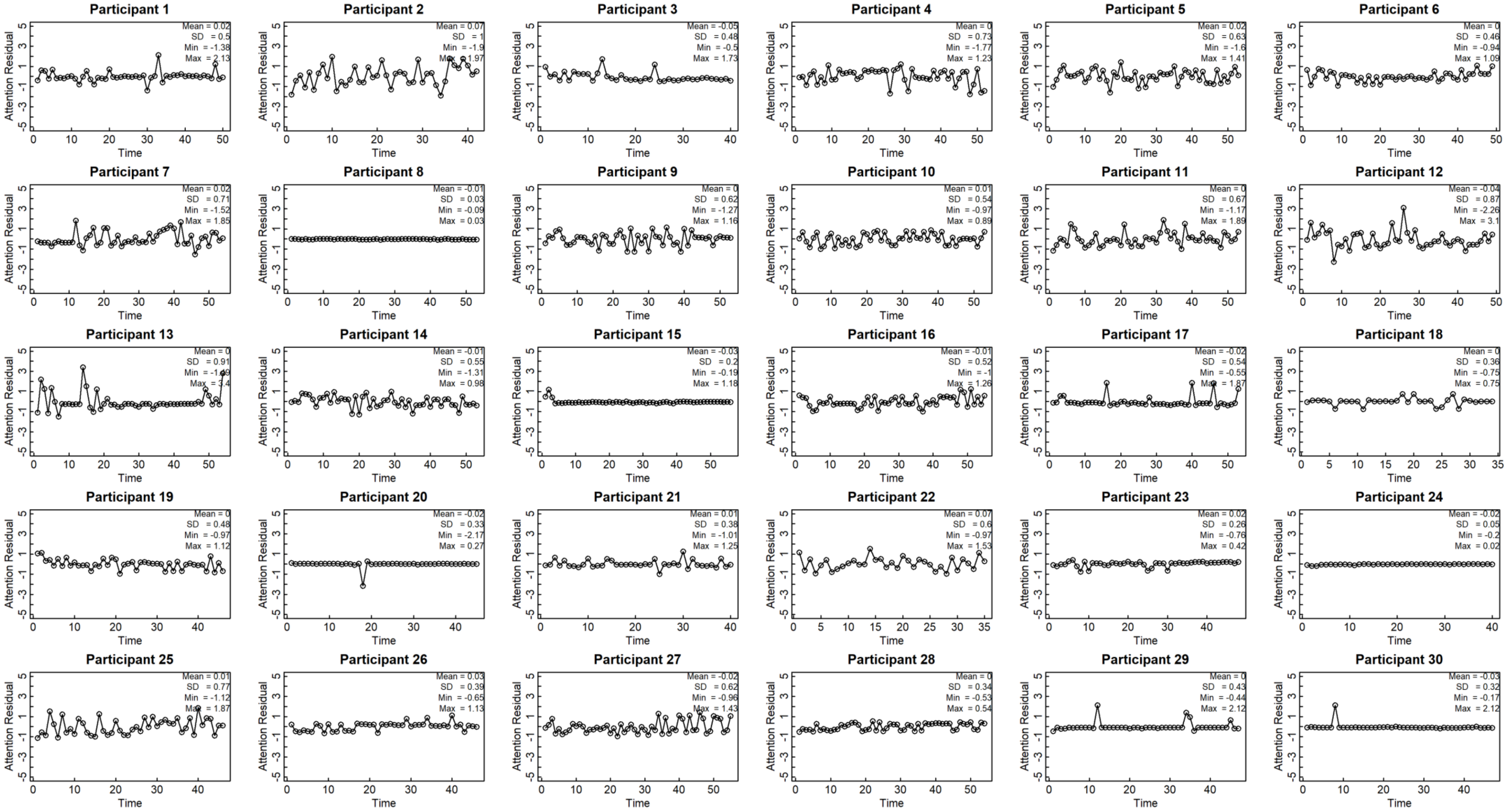

Negative affect residuals across time for each participant

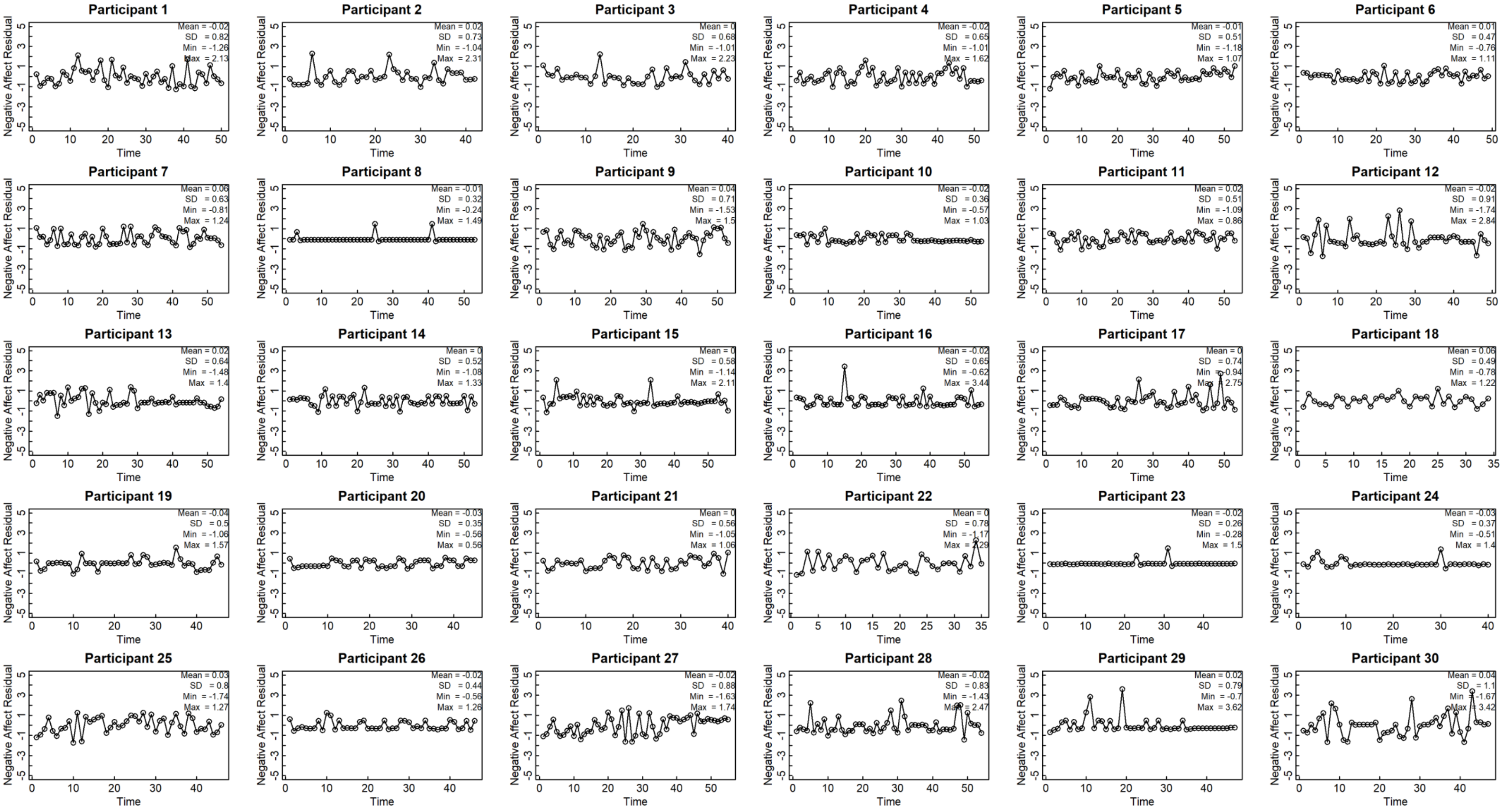

Supplement: Supplementary file 1 [file jop-167-1958-s001.pdf]
